# Management of patients with unstable angina/non-ST-elevation myocardial infarction: a critical review of the 2007 ACC/AHA guidelines

**DOI:** 10.1111/j.1742-1241.2009.01998.x

**Published:** 2009-04

**Authors:** J Hoekstra, M Cohen

**Affiliations:** 1 Department of Emergency Medicine, Wake Forest University Health ScienceWinston-Salem, NC, USA; 2 Cardiac Catheterization Laboratory, Newark Beth Israel Medical CenterNewark, NJ, USA

## Abstract

**Background:**

In 2007, the American College of Cardiology/American Heart Association (ACC/AHA) published new guidelines for the diagnosis and management of patients with unstable angina/non-ST segment elevation myocardial infarction (UA/NSTEMI). These guidelines include some important updates on the use of clopidogrel, fondaparinux, bivalirudin and low-molecular-weight heparins (LMWHs) all of which have published landmark clinical trials in patients with acute coronary syndromes (ACS) since the publication of the 2002 guidelines. While these 2007 guidelines are more comprehensive and up-to-date compared with the recommendations published in 2002, they also raise many questions for practising emergency physicians and cardiologists.

**Methods:**

This article presents a critical review of the 2007 ACC/AHA UA/NSTEMI guidelines, highlighting some of the areas of controversy, with the aim of providing some further guidance to practising physicians.

**Conclusions:**

Despite recent updates to the ACC/AHA UA/NSTEMI guidelines, additional factors need to be taken into consideration in the management of UA/NSTEMI patients. Integrating initial responses with early or selectively invasive strategies and the risks of complications in subsequent procedures require careful consideration. Protocol development within an institution is required to risk-stratify patients rapidly, provide optimum precatheterisation medical management and allow seamless and rapid transitions to the catheterisation laboratory in patients at risk for adverse events.

**Review Criteria**

- This article presents a critical review of the 2007 ACC/AHA UA/NSTEMI guideline updates.
- MEDLINE was searched in September 2007 to identify relevant clinical trials, abstracts, case reports and articles using search terms appropriate to areas of interest identified by the authors. The reference lists of pertinent articles were reviewed to identify additional publications.

**Message for the Clinic**

- Despite recent updates to the ACC/AHA guidelines for the diagnosis and management of patients with UA/NSTEMI, there are gaps in the knowledge base.
- Decisions regarding adopting an early vs. a selectively invasive strategy should only be considered after a thorough risk assessment has been performed.
- Protocol development within an institution should facilitate optimum precatheterisation medical management and allow seamless and rapid transitions to the catheterisation laboratory.

## Introduction: key differences between 2002 and 2007 guidelines

In 2007, the American College of Cardiology/American Heart Association (ACC/AHA) published new guidelines for the diagnosis and management of patients with unstable angina/non-ST-elevation myocardial infarction (UA/NSTEMI) (1). The major revisions in the 2007 guidelines (1) since the 2002 guidelines reflect the growing interest and research into improving outcomes in patients with acute coronary syndromes (ACS) (2). On patient presentation, it is important to diagnose correctly and risk-stratify according to the guidelines. Some novel biomarkers, e.g. B-type natriuretic peptide (BNP), and risk-assessment models such as the Thrombolysis In Myocardial Infarction (TIMI) score, the Global Registry of Acute Coronary Events (GRACE) or the Platelet Glycoprotein (GP) IIb/IIIa in Unstable Angina: Receptor Suppression Using Integrilin Therapy (PURSUIT) scores may be useful additions to help physicians correctly risk-stratify their patients. Secondly, the guidelines seem to encourage the use of invasive management, rather than ischaemia-guided management, although there is increasing evidence to encourage appropriate risk stratification before deciding whether patients need to be managed according to a conservative or invasive strategy. There are also new recommendations regarding the use of clopidogrel or GP IIb/IIIa inhibitors, which are incorporated in the 2007 guidelines. Furthermore, the Organisation to Assess Strategies in Acute Ischemic Syndrome (OASIS)-5 (3) and Acute Catheterisation and Urgent Intervention Triage StrategY (ACUITY) (4) trials provide new information regarding the use of fondaparinux and bivalirudin respectively in the management of patients with NSTEMI. This review not only critically evaluates new data from recent trials but also discusses the gaps in the evidence and remaining controversies in the management of patients with NSTEMI (1).

## Risk stratification: markers and tools

The 2007 ACC/AHA guideline recommendations for early risk stratification (1) remain essentially unchanged since publication of the 2002 guidelines (2). They state that patients should be stratified into one of three groups: low-, moderate- and high-risk, according to their risk factors. These risk factors include anginal symptoms, physical findings, electrocardiogram (ECG) findings and cardiac biomarkers. An early ECG, within 10 min of arrival in the emergency department, receives a class I recommendation (level of evidence: B). Previously, troponin was recommended as a very good predictor of risk (2) and the updated guidelines also mention the use of BNP as a potentially useful biomarkers for risk assessment (class IIb recommendation; level of evidence: B) (1). The TIMI or GRACE risk scores or PURSUIT risk model are recommended as useful for assisting decision-making with regard to treatment options in patients with suspected ACS (class IIa recommendation; level of evidence: B) (1). In addition, the elevated risk of bleeding and adverse events is highlighted for patients with advanced age, female sex and chronic renal insufficiency. However, the new guideline recommendations do not discuss the treatment pathways according to the patients’ risk scores (low-, moderate- and high-risk), but instead, refer to treatment decisions in the context of whether conservative or invasive management strategies are to be employed. This represents a departure from the 2002 guidelines (2) and makes upstream (i.e. before diagnostic angiography) drug treatment decisions difficult, especially when the downstream management strategy is unknown.

### Practical usage of risk-stratification tools

The three risk-stratification tools have a number of factors in common, particularly advanced age, ST-segment deviation and elevated cardiac markers. However, they are different in terms of other parameters, their practical application and which outcomes they predict. The GRACE risk model is unsuitable for risk stratification of patients for initial treatment, but can calculate the probability of in-hospital mortality (5) or 6-month postdischarge mortality (6), while the PURSUIT model predicts the rate of 30-day mortality and the composite end-point of death or myocardial (re)infarction (7). The PURSUIT and GRACE risk scores involve a complex calculation of risk, as they include both dichotomous and continuous variables, and require the use of computer-based programs based on published nomograms. A free version of the GRACE risk-assessment tool can be found online (http://www.outcomes-umassmed.org/grace/acs_risk.cfm). The TIMI risk score appears to be the simplest to remember and apply in general clinical practice. Patients are assigned a value of 1 for each prognostic variable and the patients’ total score determines their risk stratum, which is used to predict clinical outcomes and provide a basis for making treatment decisions (8) The TIMI-11B study demonstrated that the rate of all-cause mortality, myocardial infarction (MI) or severe recurrent ischaemia increased significantly as the TIMI risk score increased (p < 0.001) (8).

### Our approach

**Figure 1:**
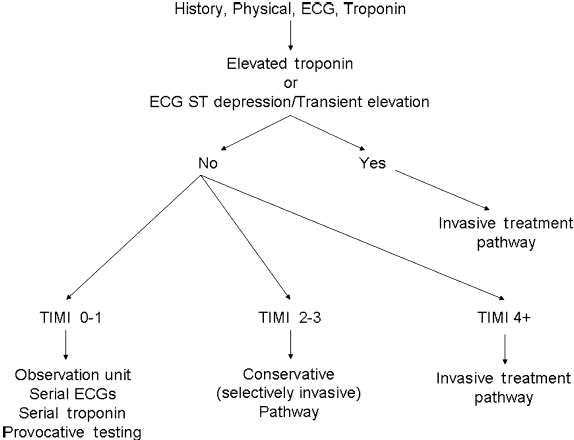
Risk-stratification flow diagram for non-ST-elevation myocardial infarction (NSTEMI) acute coronary syndrome (ACS) patients, from the Wake Forest University Health Science, Department of Emergency Medicine

At the Wake Forest University Health Science Department of Emergency Medicine, we use a combination of markers, ECG findings and risk scores to determine initial treatment and disposition. Patients with new ST depression, transient elevation or an elevated troponin are automatically considered high risk and treated via an invasive pathway according to the new guidelines. In the absence of ECG or marker changes, we use an adapted TIMI score to stratify patients into high-risk (TIMI score ≥ 4), intermediate-risk (TIMI score 2–3) or low-risk (TIMI score 0–1) ACS. Each of these groups is placed in distinct risk-matched treatment pathways with certain pharmacotherapies and diagnostic or management strategies that are predetermined and correspond to the new guideline recommendations (Figure 1).

## Risk stratification: invasive vs. conservative management

An early invasive strategy (e.g. diagnostic angiography with intent to perform revascularisation) is indicated in UA/NSTEMI patients (without serious comorbidities or contraindications to such procedures) who have: either refractory angina or haemodynamic or electrical instability (level of evidence: B) or an elevated risk for clinical events as outlined in Table 1 (level of evidence: A). A conservative (e.g. selectively invasive) strategy may be considered in initially stabilised patients (without serious comorbidities or contraindications to such procedures) who have an elevated risk for clinical events (as outlined in Table 1), including those who are troponin-positive, although this is a very weak recommendation (class IIb recommendation; level of evidence: B) (1).

**Table 1:** Selection of initial treatment strategy: invasive vs. conservative strategy

Preferred strategy	Patient characteristics
Invasive	Recurrent angina or ischaemia at rest or with low-level activities despite intensive medical therapy
	Elevated cardiac biomarkers (troponin I or T)
	New or presumably new ST-segment depression
	Signs or symptoms of heart failure or new or worsening mitral regurgitation
	High-risk findings from non-invasive testing
	Haemodynamic instability
	Sustained ventricular tachycardia
	PCI within 6 months
	Prior CABG
	High-risk score (e.g. TIMI, GRACE)
	Reduced left-ventricular function (LVEF < 40%)
Conservative	Low-risk score (e.g. TIMI, GRACE)
	Patient or physician preference in the absence of high-risk features

CABG, coronary artery bypass graft surgery; GRACE, Global Registry of Acute Coronary Events; LVEF, left-ventricular ejection fraction; PCI, percutaneous coronary intervention; TIMI, thrombolysis in myocardial infarction.

The recommendations for this predominantly invasive approach are primarily based on three large randomised trials: the Fragmin and Fast Revascularisation during Instability in Coronary Artery Disease (FRISC)-II trial (9), the Treat Angina with Aggrastat and Determine Cost of Therapy with an Invasive or Conservative Strategy–Thrombolysis in Myocardial Infarction 18 (TACTICS–TIMI-18) trial (10) and the third Randomised Intervention Treatment of Angina (RITA 3) trial (11). These studies showed that an early invasive strategy was beneficial for preventing ischaemic outcomes, especially in subgroups of patients at high risk, such as those presenting with an elevated cardiac troponin level. For example, one study showed that the odds ratio (OR) for death, non-fatal MI or re-hospitalisation was 0.44 [95% confidence interval (CI) 0.30–0.66] at 30 days and 0.55 (95% CI 0.40–0.75) at 6 months in patients with elevated troponin levels (10). These findings were confirmed in a meta-analysis of trials comparing a routine invasive strategy with a more conservative strategy in patients with NSTEMI. This meta-analysis showed that routine invasive strategies were more effective in preventing MI (7.3% vs. 9.4%, OR 0.75, 95% CI 0.65–0.88), severe angina (11.2% vs. 14.0%, OR 0.77, 95% CI 0.68–0.87) and re-hospitalisation (32.5% vs. 41.3%, OR 0.66, 95% CI 0.60–0.72) when compared with a conservative strategy (12).

Historically, an ischaemia-guided approach was adopted in patients presenting with suspected cases of UA/MI. In this strategy, patients only receive diagnostic cardiac catheterisation and revascularisation if myocardial ischaemia has been objectively diagnosed, such as through recurrent symptoms or provocative stress testing. However, as early trial data increasingly supported better outcomes with an ‘early invasive’ strategy (9–11), there has been a decline in ischaemia-guided medical management. Accordingly, patients presenting with elevated risk for adverse outcomes from ACS are now routinely referred for early coronary angiography and increasingly undergo percutaneous coronary intervention (PCI).

**Figure 2:**
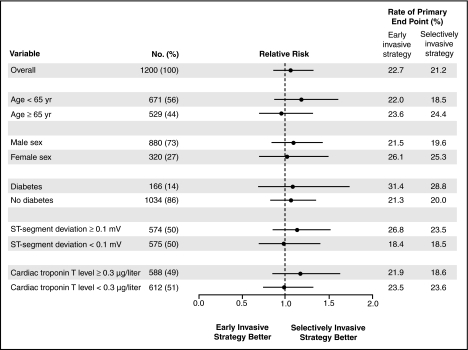
Comparison of primary outcome (composite of death, non-fatal myocardial infarction, or re-hospitalisation for anginal symptoms within 1 year) with an early invasive vs. a selectively invasive strategy in acute coronary syndromes (ACS) (reproduced with permission from de Winter, et al.; Invasive vs. Conservative Treatment in Unstable Coronary Syndromes (ICTUS) Investigators. Early invasive vs. selectively invasive management for acute coronary syndromes. *N Engl J Med* 2005; **353:** 1095–1104) (13). © 2005 Massachusetts Medical Society. All rights reserved

However, not all studies support an early invasive strategy for all patients. For example, the Invasive vs. Conservative Treatment in Unstable Coronary Syndromes (ICTUS) trial compared an early invasive to a selectively invasive strategy using low-molecular-weight heparin (LMWH), GP IIb/IIIa inhibition (clopidogrel) and intensive lipid-lowering therapy in high-risk UA/NSTEMI patients (with elevated cardiac troponin T levels) (13). The selectively invasive strategy was associated with similar outcomes compared with early invasive strategy, across a spectrum of high-risk patients (Figure 2) (13). These outcomes were consistently observed in both the short- (13) and long-term (14). It should be noted that the medical management in ICTUS was very aggressive and that a high percentage of patients in the ‘conservative’ arm underwent early revascularisation.

Furthermore, there is conflicting evidence from the FRISC-II and RITA 3 trials regarding the long-term benefits of an early invasive strategy (15–17). The FRISC-II trial found that early invasive management was associated with a significant reduction in mortality at 2 years [3.7% vs. 5.4%, relative risk (RR) 0.68, 95% CI 0.47–0.98, p = 0.04] (15), but that this benefit was not sustained at 5 years (9.7% vs. 10.1%, RR 0.95, 95% CI 0.75–1.21, p = 0.69) (16). In the RITA 3 study, the survival curves started to diverge in favour of the early invasive strategy only after 2 years, resulting in a mortality of 12.1% with the early invasive strategy and 15.1% with the conservative strategy at 5 years (OR 0.76, 95% CI 0.58–1.00, p = 0.054) (17).

Further consideration needs to be given to the patient groups for whom the intensive nature of an invasive upstream treatment strategy may not be appropriate, e.g. in women with low-risk features, the extremely elderly, patients presenting with severe renal dysfunction and those patients who have previously undergone coronary bypass surgery or PCI (1).

Studies have shown that event rates increase significantly with each additional risk factor (p < 0.001 for trend) (8) and that the greatest clinical benefits are seen when patients are managed according to their individual risk (8,10,18). Patients who receive the most benefit from the early invasive approach appear to be those with elevated troponin levels and/or TIMI risk scores of ≥ 4. Collectively, these data suggest that efforts should focus on providing invasive strategies to patients who would benefit most from these interventions and therefore, risk stratification of patients with UA/NSTEMI is very important before deciding on a future management approach.

## Use of GP IIb/IIIa inhibitors and/or clopidogrel

According to the ACC/AHA guidelines (1), antiplatelet therapy in addition to aspirin should be initiated before diagnostic angiography (upstream), with either clopidogrel (loading dose followed by daily maintenance dose) or an intravenous (i.v.) GP IIb/IIIa inhibitor (class I recommendation; level of evidence: A) (1). Clopidogrel can also be used in conjunction with i.v. GP IIb/IIIa inhibitor (class IIa recommendation; level of evidence: B). Factors favouring administration of both agents include: delay to angiography, high-risk features such as elevated troponin and early recurrent ischaemic discomfort. These two recommendations imply that the treatment approach may vary according to the patient’s characteristics.

There is good evidence that treatment with clopidogrel prior to PCI prevents postprocedural ischaemic complications (19,20). The First Intracoronary Stenting and Antithrombotic Regimen: Rapid Early Action for Coronary Treatment (ISAR-REACT) trial investigated the use of a 600 mg loading dose of clopidogrel with or without GP IIb/IIIa inhibitors in low-risk patients undergoing PCI (21). Clopidogrel alone was well tolerated and associated with a low frequency of early complications, but there was no additional clinically measurable benefit at 30 days with the administration of the GP IIb/IIIa inhibitor, abciximab, in this low-risk cohort (21).

Other studies have shown that the most benefit from GP IIb/IIIa inhibitors is observed in patients at intermediate-to-high risk requiring PCI (22–27). For example, among high-risk patients with an elevated troponin level, the ISAR-REACT 2 study found that the incidence of events was significantly lower with concurrently administered clopidogrel and abciximab (67/513 patients; 13.1%) than clopidogrel and placebo (98/536 patients; 18.3%). This corresponded to an RR of 0.71 (95% CI 0.54–0.95, p = 0.02) (p = 0.07 for the interaction) (28). These results support the utilisation of clopidogrel and GP IIb/IIIa inhibitors in high-risk NSTEMI ACS patients, especially those with elevated troponin levels.

However, the benefits of these treatments in certain subgroups of patients, such as the elderly and patients with renal impairment, are less certain. For instance, reports on the efficacy of GP IIb/IIIa inhibitors in the elderly show apparently contradictory results (29–31). One study reported a reduction in major ischaemic events associated with GP IIb/IIIa inhibitors in elderly patients (10% vs. 5.9%, OR 0.56, 95% CI 0.30–0.83) (29), whereas the other showed no significant differences (9.9% vs. 10.9%, RR 1.10, 95% CI 0.72–1.69, p = 0.65) (30). Furthermore, the suitability of using concomitant GP IIb/IIIa inhibitors and clopidogrel in these potentially high-risk patient subgroups is yet to be established.

In summary, the findings from these trials reinforce the need for careful evaluation of risks, clinical features and patient characteristics most associated with benefits when selecting therapeutic regimens (32). Risk stratification should enable physicians to select which patients should receive clopidogrel alone or clopidogrel in combination with GP IIb/IIIa inhibitors.

## Pretreatment with clopidogrel prior to invasive procedures

Clopidogrel is recommended by the ACC/AHA guidelines either during an invasive or conservative approach (class IA recommendation; level of evidence: A) (1). In the PCI Clopidogrel in Unstable Angina to Prevent Recurrent Events (CURE) study, the frequency of death or MI in the 30 days after PCI was significantly lower among patients who had been pretreated with clopidogrel for a median of 10 days compared with those patients who received no pretreatment (4.4% vs. 2.8%, relative risk reduction 34%, p < 0.04) (33). There was no significant increase in major or minor bleeding associated with clopidogrel pretreatment in patients who underwent a percutaneous revascularisation (33). On the basis of this study, it seems that early treatment with clopidogrel reduces early ischaemic events.

However, the use of clopidogrel may not be appropriate for all patients. Between 12% and 27% of patients requiring coronary revascularisation undergo coronary artery bypass grafting (CABG) as their primary mode of therapy (9,10,34,35). Some studies suggest that the use of clopidogrel pretreatment may increase the risk of 30-day any major bleeding when compared with no pretreatment (2.0% vs. 1.5%, RR 1.31, 95% CI 1.01–1.70) (36). As bleeding represents a serious complication for CABG, early risk stratification is key to the identification of those who may need urgent CABG and who should not receive clopidogrel. This risk-stratification strategy will minimise the bleeding risk for those proceeding to CABG, while ensuring that patients undergoing PCI benefit from clopidogrel treatment (37).

## Use of antithrombins in the invasive strategy

The key studies of antithrombin therapy in NSTEMI patients are summarised in Table 2 (4,38–48). The ACC/AHA guidelines recommend the use of enoxaparin or unfractionated heparin (UFH) as antithrombin therapy in the invasive pathway (class IA recommendation; level of evidence: A) (1). The ACC/AHA guidelines also recommend fondaparinux during an invasive approach (class IB recommendation; level of evidence: B). The current evidence indicates that fondaparinux and UFH have comparable clinical safety in patients undergoing PCI in both NSTEMI (12,48) and STEMI (47) patients. However, there was a higher rate of guiding catheter thrombosis with fondaparinux in the OASIS-5 trial (3). After the OASIS-5 protocol was amended to include administration of i.v. UFH to patients undergoing PCI, the rate of this complication was lower (3). However, the exact dose of UFH needed to prevent catheter-thrombosis formation during the use of fondaparinux in patients undergoing PCI remains undefined. This confusion regarding heparin dosing in the catheterisation laboratory in patients already on fondaparinux has led to slow acceptance of fondaparinux in high-risk patients.

**Table 2:** Key studies in non-ST-elevation myocardial infarction patients (4,38–48)

					Efficacy results	Safety results
Clinical trial (reference)	Patients, *n*	Test drug	Comparator drug	End-point†	End-point incidence and analyses	Major bleeding
ACUITY (4)	13,819	Bivalirudin i.v. 0.1 mg/kg bolus then infusion of 0.25 mg/kg/h. i.v. 0.5 mg/kg bolus before PCI, then infusion increased to 1.75 mg/kg/h	GP IIb/IIIa antagonists: eptifibatide i.v. 180 μg/kg bolus plus 2.0 μg/kg/min infusion) or tirofiban: 0.4 μg/kg/min infusion for 30 min followed by 0.1 μg/kg/min infusion or abciximab: 0.25 mg/kg bolus plus 0.125 μg/kg/min infusion, max 10 μg/min plus either UFH 60 U/kg i.v. bolus followed by i.v. infusion of 12 U/kg/h adjusted for aPTT, or enoxaparin 1 mg/kg SC every 12 h	Death, MI or urgentrevascularisation at 30 days	Bivalirudin 7.8% vs. heparin 7.3%; RR 1.08, 95% CI 0.93 to 1.24; p = 0.32	Bivalirudin 3% vs. heparin 5.7%; RR 0.53; p < 0.001
		As above plus GP IIb/IIIa antagonists: eptifibatide i.v. 180 μg/kg bolus plus 2.0 μg/kg/min infusion) or tirofiban: 0.4 μg/kg/min infusion for 30 min followed by 0.1 μg/kg/min infusion or abciximab: 0.25 mg/kg bolus plus 0.125 μg/kg/min infusion, maximum 10 μg/min			Heparin 7.3% vs. bivalirudin plus GP IIb/IIIa antagonists 7.7%; RR 1.07; 95% CI 0.92 to 1.23; p = 0.39	Heparin 5.7% vs. bivalirudin plus GP IIb/IIIa antagonists 5.3% RR 0.93; p < 0.001
FRISC (38)	1506	Dalteparin 120 IU/kg* SC bid (maximum 10,000 IU) for 6 days	Placebo	Death or new MI at day 6	Dalteparin 1.8% vs. placebo 4.8%; RR 0.37; ARR 3%; 95% CI 0.20 to 0.68; p = 0.001	Dalteparin 0.8% vs. placebo 0.5%; ARR 0.3%
		As above then dalteparin 7500 IU SC once daily for 35–45 days		Death or new MI at day 40	Dalteparin 8% vs. placebo 10.7%; RR 0.75; ARR 2.7%; 95% CI 0.54 to 1.03; p = 0.07	Dalteparin 0.3% vs. placebo 0.3%; ARR 0%
ESSENCE (39)	3171	Enoxaparin 1 mg/kg SC bid minimum 48 h, maximum 8 days	UFH i.v. bolus (5000 U) and continued i.v. infusion	Death, MI, or recurrent angina at 14 days	Enoxaparin 16.6% vs. UFH 19.8%; OR 0.80; ARR 3.2%; 95% CI 0.67 to 0.96; p = 0.019	
				Death, MI, or recurrent angina at 30 days	Enoxaparin 19.8% vs. UFH 23.3%; OR 0.81; ARR 3.5%; 95% CI 0.68 to 0.96; p = 0.016	Enoxaparin 6.5% vs. UFH 7%; ARR 0.5%; p = 0.57
FRIC (40)	1482	Dalteparin 120 IU/kg SC bid for 6 days	UFH 5000 U i.v. bolus and i.v. infusion of 1000 U/h for 48 h	Death, MI, or recurrence of angina	Dalteparin 9.3% vs. UFH 7.6%; RR 1.18; ARR −1.7%; 95% CI 0.84 to 1.66; p = 0.33	Dalteparin 1.1% vs. UFH 1.0%; ARR −0.1%
				Death or MI	Dalteparin 3.9% vs. UFH 3.6%; RR 1.07; ARR −0.3%; 95% CI 0.63 to 1.80; p = 0.80	
		Dalteparin 7500 IU SC once per day between days 6 and 45	Placebo SC once daily	Death, MI, or recurrence of angina	Dalteparin 12.3% vs. UFH 12.3%; RR 1.01; ARR 0%; 95% CI 0.74 to1.38; p = 0.96	Dalteparin 0.5% vs. placebo 0.4%; ARR −0.1%
				Death or MI	Dalteparin 4.3% vs. placebo 4.7%; RR 0.92; ARR 0.4%; 95% CI 0.54 to 1.57; p = 0.76	
FRAXIS (41)	3468	Nadroparin 86 anti-Xa IU/kg i.v. bolus, followed by nadroparin 86 anti-Xa IU/kg SC bid for 6 days (plus or minus 2 days)	UFH 5000 U i.v. bolus and UFH infusion at 1250 U/h i.v. for 6 days (plus or minus 2 days)	Cardiac death, MI, refractory angina, recurrence of UA at day 14	Nadroparin 17.8% vs. UFH 18.1%; ARR 0.3%; 95% CI −2.8 to 3.4; p = 0.85	Nadroparin 1.5% vs. UFH 1.6%; ARR 0.1%
		Nadroparin 86 anti-Xa IU/kg i.v. bolus, followed by nadroparin 86 anti-Xa IU/kg SC bid for 14 days			Nadroparin 20.0% vs. UFH 18.1%; ARR −1.9%; 95% CI −5.1 to 1.3; p = 0.24	Nadroparin 3.5% vs. UFH 1.6%; ARR −1.9%; p = 0.0035
TIMI 11B (42)	3910	Inpatient: enoxaparin 30 mg i.v. bolus immediately followed by 1 mg/kg SC every 12 h	UFH 70 U/kg bolus and infusion at 15 U/h titrated to aPTT (treatment maintained for a minimum of 3 and maximum of 8 days at physician’s discretion)	Death, MI, urgent revascularisation at 48 h	Enoxaparin 5.5% vs. UFH 7.3%; OR 0.75; ARR 1.8%; 95% CI 0.58 to 0.97; p = 0.026	Enoxaparin 0.8% vs. UFH 0.7%; ARR −0.1%; p = 0.14
				Death, MI, urgent revascularisation at 8 days	Enoxaparin 12.4% vs. UFH 14.5%; OR 0.83; ARR 2.1%; 95% CI 0.69 to 1.00; p = 0.048	End of initial hospitalisation: enoxaparin 1.5% vs. UFH 1%; ARR −0.5%; p = 0.143
		Outpatient: enoxaparin 40 mg SC bid (patients weighing < 65 kg) or 60 mg SC bid patients weighing at least 65 kg)	Placebo SC bid	Death, MI, urgent revascularisation at14 days	Enoxaparin 14.2% vs. UFH 16.7%; OR 0.82; ARR 2.5%; 95% CI 0.69 to 0.98; p = 0.029	
				Death, MI, urgent revascularisation at 43 days	Enoxaparin 17.3% vs. UFH 19.7%; OR 0.85; ARR 2.4%; 95% CI 0.72 to 1.00; p = 0.048	Between days 8 and 43: enoxaparin 2.9% vs. placebo 2.9%; ARR 0%; p = 0.021
ACUTE II (43)	525†	Enoxaparin 1 mg/kg SC every 12 h	UFH 5000 U i.v. bolus and maintenance infusion at 1000 U/h i.v. adjusted to aPTT	Death at 30 days	Enoxaparin 2.5% vs. UFH 1.9%, RR −1.3; ARR −0.6%; 95% CI 0.06 to 3.93; p = 0.77	Enoxaparin 0.3% vs. UFH 1%; ARR 0.7%; p = 0.57
INTERACT (44)	746‡	Enoxaparin 1 mg/kg SC every 12 h	UFH 70 U/kg i.v. bolus followed by continuous infusion at 15 U/kg/h	Death or MI at 30 days	Enoxaparin 5.0% vs. UFH 9.0%; RR 0.55; ARR 4%, 95% CI 0.30 to 0.96; p = 0.031	At 96 h: enoxaparin 1.8% vs. UFH 4.6%; ARR 2.8%; p = 0.03
A to Z (45)	3987§	Enoxaparin 1 mg/kg SC every 12 h	Patients ≥ 70 kg: UFH 4000 U i.v. bolus followed by 900 U/h i.v. infusion; Patients ≤ 70 kg UFH 60 U/kg (maximum 4000 U) i.v. bolus followed by 12 U/kg/h i.v. infusion	All-cause death, MI, or refractory ischaemia within 7 days of tirofiban initiation	Enoxaparin 8.4% vs. UFH 9.4%; HR 0.88; ARR 1%; 95% CI 0.71 to 1.08	Enoxaparin 0.9% vs. UFH 0.4%; ARR −0.5%; p = 0.05
SYNERGY¶ (46)	9978	Enoxaparin 1 mg/kg SC every 12 h	UFH 60 U/kg i.v. bolus (maximum of 5000 U) and followed by i.v. infusion of 12 U/kg/h (maximum of 1000 U/h initially	Death or non-fatal MI during first 30 days	Enoxaparin 14.0% vs. UFH 14.5%; HR 0.96; ARR 0.5%; 95% CI 0.86 to 1.06; p = 0.40	Enoxaparin 9.1% vs. UFH 7.6%; ARR −1.5%; p = 0.008
ASPIRE (47)	350	Fondaparinux i.v. 2.5 mg	UFH (100 U/kg without GP IIb/IIIa antagonist, or 65 U/kg with GP IIb/IIIa antagonist as per local practice)	Death, MI, urgent revascularisation, or bailout use of GP IIb/IIIa antagonist	Fondaparinux 4.2% vs. UFH 6.0%	Fondaparinux 0.8% vs. UFH 0.0%
		Fondaparinux i.v. 5.0 mg			Fondaparinux 7.8% vs. UFH 6.0%	Fondaparinux 2.6% vs. UFH 0.0%
		Either 2.5 or 5.0 mg fondaparinux i.v.			Fondaparinux 6.0% vs. UFH 6.0%; HR 1.01; 95% CI 0.41 to 2.52; p = 0.97	Total bleeding: fondaparinux 6.4% vs. UFH 7.7%; HR 0.81; 95% CI 0.35 to 1.84; p = 0.61
OASIS-5(Yusuf(et al.,2006) (48)	20,078	Fondaparinux 2.5 mg/kg SC once daily	Enoxaparin 1 mg/kg SC every 12 h	Death, MI, or refractory ischaemia 9 days	Fondaparinux 5.8% vs. enoxaparin 5.7%; HR 1.01; 95% CI 0.90 to 1.13; p = 0.007	Fondaparinux 2.2% vs. enoxaparin 4.1%; p < 0.001
				Death, MI, or refractory ischaemia 30 days	Fondaparinux 8.0% vs. enoxaparin 8.6%; HR 0.93; 95% CI 0.84 to 1.02; p = 0.13	
				Death, MI, or refractory ischaemia 180 days	Fondaparinux 12.3% vs. enoxaparin 13.2%; HR 0.93; 95% CI 00.86 to 1.00; p = 0.06	

End-point timings same as end of treatment period unless stated otherwise. Partially based on data from ACC/AHA 2007 Guidelines for the Management of Patients with Unstable Angina/Non-ST-Elevation Myocardial Infarction (1). For specific interventions and additional medications during the study, see individual study references. Major bleeding was classified as follows in the various trials: ACUITY: major bleeding was defined as the cumulative occurrence within 25 to 35 days after randomisation of intracranial or intra-ocular bleeding, haemorrhage at the access site requiring intervention, haematoma with a diameter of at least 5 cm, a reduction in haemoglobin levels of at least 4 g/dl without an overt bleeding source or at least 3 g/dl with such a source, reoperation for bleeding, or transfusion of a blood product. FRISC: major bleeding was defined as a fall in haemoglobin of more than 20 g/l (2 g/dl) associated with corresponding signs or symptoms, intracranial bleeding, or bleeding leading to transfusion, interruption of treatment, or death. ESSENCE: major haemorrhage was defined as bleeding resulting in death, transfusion of at least 2 U of blood, a fall in haemoglobin of 30 g/l or more, or a retroperitoneal, intracranial, or intra-ocular haemorrhage. FRIC: a bleeding event was classified as major if it led to a fall in the haemoglobin level of at least 20 g/l, required transfusion, was intracranial, or caused death or cessation of the study treatment. FRAXIS: haemorrhage was considered as major if it met any of the following criteria: symptomatic bleeding associated with a decrease in haemoglobin > 2 g/dl; retroperitoneal or intracranial haemorrhage; haemorrhage requiring transfusion or haemorrhagic death. TIMI 11B: overt bleed resulting in death; a bleed in a retroperitoneal, intracranial, or intra-ocular location; a haemoglobin drop of greater than or equal to 3 g/l; or the requirement of transfusion of at least 2 U of blood. SYNERGY: TIMI and GUSTO criteria. ACUTE II: severity was recorded on the basis of the TIMI trial bleeding criteria. TIMI major bleeding involved a haemoglobin drop greater than 5 g/dl (with or without an identified site, not associated with coronary artery bypass grafting) or intracranial haemorrhage or cardiac tamponade. INTERACT: major bleeding included bleeding resulting in death, or retroperitoneal haemorrhage, or bleeding at a specific site accompanied by a drop in haemoglobin greater than or equal to 3 g/dl. A to Z: decrease in haemoglobin of more than 5 mg/dl or intracranial or pericardial bleeding. SYNERGY: TIMI and GUSTO definitions. ASPIRE: major bleeding was defined as clinically overt bleeding with one of the following criteria: fatal, symptomatic intracranial haemorrhage, retroperitoneal haemorrhage, intra-ocular haemorrhage, or a fall in haemoglobin of 3.0 g/dl, with each blood transfusion unit counting for 1.0 g/dl of haemoglobin, or transfusion of ≥ 2 U of blood. OASIS-5: major bleeding is defined as clinically overt bleeding that is either fatal, symptomatic intracranial, retroperitoneal, intra-ocular, a decrease in haemoglobin of at least 3.0 g/dl (with each blood transfusion unit counting for 1.0 g/dl of haemoglobin), or requiring transfusion of ≥ 2 U of red blood cells. *Initial trial dose of 150 IU/kg SC bid decreased to 120 IU/kg SC bid due to increased bleeding during first 6 days (four patients or 6% major bleeding episodes and nine patients or 14% minor episodes among 63 actively treated patients). †All patients in ACUTE II received a tirofiban loading dose of 0.4 μg/kg/min over 30 min, followed by a maintenance infusion at 0.1 μg/kg/min. ‡All patients in INTERACT received eptifibatide 180 μg/kg bolus followed by a 2.0 μg/kg/min infusion for 48 h. §All patients enrolled in the A to Z Trial received aspirin and tirofiban. ¶Patients also received glycoprotein IIb/IIIa inhibitors, aspirin, clopidogrel; patients eligible for enrolment even if LMWH or UFH given before enrolment, adjustments made to enoxaparin and UFH during percutaneous coronary intervention. A to Z, Aggrastat to Zocor study; ACUITY, Acute Catheterisation and Urgent Intervention Triage strategY trial; ACUTE II, antithrombotic combination using tirofiban and enoxaparin; aPTT, activated partial thromboplastin time; ARR, absolute risk reduction; ASPIRE, Arixtra Study in Percutaneous coronary Intervention: a Randomised Evaluation; bid, twice daily; CI, confidence interval; ESSENCE, efficacy and safety of subcutaneous enoxaparin in unstable angina and non-Q-wave myocardial infarction; FRAXIS, fraxiparine in ischaemic syndrome; FRIC, fragmin in unstable coronary disease; FRISC, fragmin during instability in coronary artery disease; HR, hazard ratio; INTERACT, integrilin and enoxaparin randomised assessment of acute coronary syndrome treatment; IU, international units; i.v., intravenous; LD, loading dose; LMWH, low-molecular-weight heparin; MD, maintenance dose; MI, myocardial infarction; *n*, number of patients; OASIS-5, Organisation to Assess Strategies in Acute Ischemic Syndromes-5 trial; RR, relative risk; SC, subcutaneous; SYNERGY, superior yield of the new strategy of enoxaparin, revascularisation and glycoprotein IIb/IIIa inhibitors; TIMI 11B, thrombolysis in myocardial infarction 11B; U, unit; UA, unstable angina; UFH, unfractionated heparin.

On the basis of findings from OASIS-5 in NSTEMI patients, the guidelines also recommend upstream therapy with fondaparinux in patients managed conservatively with a high risk of bleeding (class IB recommendation; level of evidence: B). This recommendation for the broader application of fondaparinux in patients at high risk of bleeding was made despite the lack of direct evidence for an association between patient characteristics and bleeding rates from the OASIS-5 trial (3).

Hence, although the study data on fondaparinux are generally robust, there are various details missing from the published literature with fondaparinux and further information is required to help guide the ‘interventionalists’ in this situation. The manufacturers of fondaparinux have applied for US Food and Drug Administration (FDA) approval for an indication for use in ACS.

## Use of upstream bivalirudin

Bivalirudin is indicated as an antithrombin therapy in patients undergoing an invasive strategy (class IB recommendation; level of evidence: B). The use of upstream bivalirudin is also indicated as an alternative to GP IIb/IIIa inhibitors (class IIa recommendation; level of evidence: B) (1), but only with the concomitant use of clopidogrel. However, there are limited data on upstream use of bivalirudin. The use of bivalirudin monotherapy, bivalirudin plus GP IIb/IIIa inhibitors and heparin plus GP IIb/IIIa inhibitors was investigated in the ACUITY trial (Table 2) (4). The 30-day rates of composite ischaemic end-point of death, MI, or unplanned revascularisation for ischaemia were 7.7%, 7.3% and 7.8% for bivalirudin plus GP IIb/IIIa inhibitors, heparin plus GP IIb/IIIa inhibitors and bivalirudin monotherapy respectively. The RR (95% CI) for the comparison between heparin plus GP IIb/IIIa inhibitors vs. bivalirudin plus GP IIb/IIIa inhibitors was 1.01 (0.90–1.12) and for the comparison between heparin plus GP IIb/IIIa inhibitors and bivalirudin alone, 1.08 (0.93–1.24). The rates of major bleeding for the same groups were 5.3%, 5.7% and 3.0% respectively and the RRs (95% CIs) were 0.93 (0.78–1.10) and 0.53 (0.43–0.65) respectively (4). These data suggest that bivalirudin alone could be used to achieve similar efficacy and a reduction in major bleeding. However, the utilisation of bivalirudin upstream in the ACUITY trial was relatively short, with a median infusion duration of < 6 h. In addition, the majority of patients in ACUITY had been pretreated with heparin or enoxaparin prior to randomisation. As such, it is unclear how effective bivalirudin alone is in prolonged upstream precatheterisation medical management.

In addition, other treatment options should not be excluded for high-risk patients. In particular, the use of GP IIb/IIIa inhibitors should be considered for those with positive troponin and for those not pretreated with thienopyridine. Bivalirudin monotherapy in patients with positive troponin and for those not pretreated with thienopyridine was associated with an increased RR for ischaemic events, 1.12 (95% CI 0.94–1.34) and 1.29 (95% CI 1.03–1.63) respectively (4,49). Currently, bivalirudin remains an unapproved and expensive treatment option outside the catheterisation laboratory, with limited data on upstream effectiveness.

## Putting it all together: matching treatment to risk

The ACC/AHA guideline recommended therapies for patients with UA/NSTEMI are best utilised in a risk-matched strategy, which couples high-intensity treatment with high-risk patients and lower-intensity treatment with lower-risk patients. Matching treatment to risk is easily accomplished by utilising the invasive pathway for the highest-risk patients and the conservative pathway for lower-risk patients and starting this differentiation as early as the emergency department.

**Figure 3:**
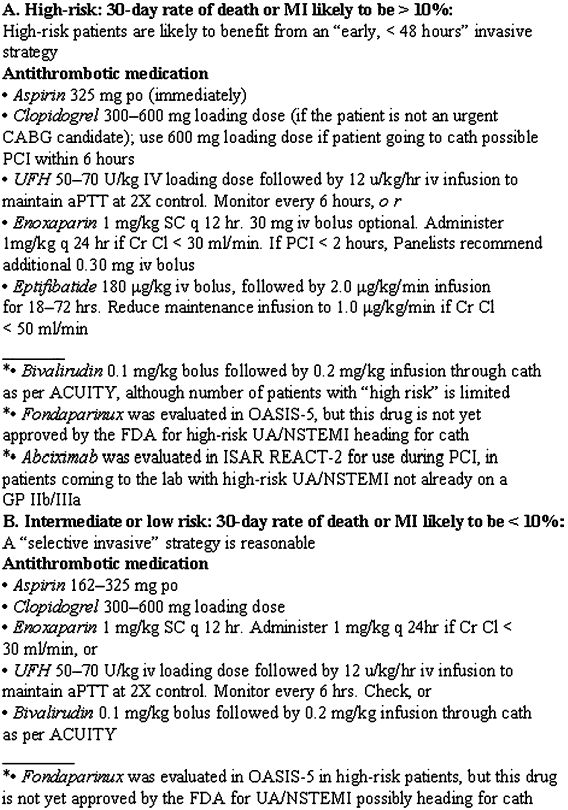
Risk-stratification treatment strategy for the management of non-ST-elevation myocardial infarction (NSTEMI)-acute coronary syndrome (ACS) (used with permission from Cohen, et al. Strategies for optimizing outcomes in the NSTE-ACS patient The CATH (cardiac catheterization and antithrombotic therapy in the hospital) Clinical Consensus Panel Report. *J Invasive Cardiol* 2006; **18:** 617–39) (50)

Thus, as mentioned above, patients with ECG ST deviation or elevated troponins or a TIMI score > 3 should be treated upstream as per the invasive pathway (Figure 3) (50). Invasive pathway medications include anti-ischaemics [oxygen, nitrates, beta-blockers and angiotensin-converting enzyme (ACE) inhibitors], aspirin, either clopidogrel or a GP inhibitor and an antithrombin (UFH or enoxaparin). An alternative to heparins plus a GP inhibitor is bivalirudin administered upstream with concomitant clopidogrel. In higher-risk patients, with elevated troponin, recurrent ischaemia on therapy or delay to catheterisation, triple antiplatelet therapy with aspirin, clopidogrel and a GP inhibitor is an alternative strategy.

In lower-risk patients, with non-diagnostic ECGs, normal troponins and TIMI scores of 2–3, the conservative or selectively invasive strategy is preferable (Figure 3) (50). Other patients who would fit in this strategy are high-risk patients who are not eligible for catheterisation for reasons of lack of capabilities, patient preference and physician preference. Conservative pathway treatments include anti-ischaemic therapy, aspirin, clopidogrel and an antithrombin or factor Xa inhibitor such as fondaparinux. LMWHs such as enoxaparin are preferable to UFH in these medically-managed patients. Patients in the conservative pathway are admitted to the hospital for serial ECGs, serial troponins and provocative testing. Catheterisation is reserved for those patients who develop recurrent chest pain, ECG changes, elevated troponins, high-risk provocative testing results or a left-ventricular ejection fraction (LVEF) < 40%. If these complications develop, then the patient is transferred to the invasive pathway and cardiac catheterisation is performed.

## Conclusions

Although the ACC/AHA guidelines are a comprehensive tool for the management of patients with NSTEMI, additional factors need to be taken into consideration to aid the decision-making process. Rapid and accurate risk stratification is essential to determine whether to use an early or selectively invasive strategy. Consideration of the risk of complications during subsequent procedures, e.g. catheter thrombosis during PCI with fondaparinux and bleeding with clopidogrel during CABG, are also important determinants for the choice of treatment. Finally, upstream medical management should be matched with catheter-based therapies to ensure seamless transitions from the precatheterisation medical management phase of therapy to the catheterisation laboratory. Although the new ACC/AHA guidelines provide many options for both precatheterisation medical management as well as catheter-based therapy, they do not provide the guidance needed to facilitate transition of care in a way that matches treatment to risk. Protocol development within an institution is required to risk-stratify patients rapidly, provide optimum precatheterisation medical management and allow seamless and rapid transitions to the catheterisation laboratory in patients at risk for adverse events.
